# Modulating neurotoxicity through CX3CL1/CX3CR1 signaling

**DOI:** 10.3389/fncel.2014.00229

**Published:** 2014-08-08

**Authors:** Cristina Limatola, Richard M. Ransohoff

**Affiliations:** ^1^Department of Physiology and Pharmacology, Istituto Pasteur Fondazione Cenci Bolognetti, Sapienza University of RomeRome, Italy; ^2^Istituto di Ricovero e Cura a Carattere Scientifico Neuromed, Istituto Neurologico MediterraneoPozzilli, Italy; ^3^Neuroinflammation Research Center, Lerner Research Institute and Cleveland Clinic Lerner College of Medicine Case Western Reserve UniversityCleveland, OH, USA

**Keywords:** CX3CL1, CX3CR1, microglia, neurotoxicity, signaling

## Abstract

Since the initial cloning of fractalkine/CX3CL1, it was proposed that the only known member of the CX3C or δ subfamily of chemotactic cytokines could play some significant role in the nervous system, due to its high expression on neurons. The pivotal description of the localization of the unique CX3CL1 receptor, CX3CR1, on microglial cells, firmed up by the generation of *cx3cr1^GFP/GFP^* mice, opened the road to the hypothesis of some specific key interactions between microglia and neurons mediated by this pair. This expectation has been indeed supported by recent exciting evidence indicating that CX3CL1-mediated microglia-neuron interaction modulates basic physiological activities during development, adulthood and aging, including: synaptic pruning; promoting survival of neurons and neural precursors; modulating synaptic transmission and plasticity; enhancing synapse and network maturation; and facilitating the establishment of neuropathic pain circuits. Beyond playing such fascinating roles in physiological conditions, CX3CL1 signaling has been implicated in different neuropathologies. Early papers demonstrated that the levels of CX3CL1 may be modulated by various toxic stimuli *in vitro* and that CX3CL1 signaling is positively or negatively regulated in EAE and MS, in HIV infection and LPS challenge, in epilepsy, in brain tumors, and in other neuropathologies. In this review we focus on the experimental evidence of CX3CL1 involvement in neuroprotection and survey the common molecular and cellular mechanisms described in different brain diseases.

The CX3CL1/CX3CR1 axis, together with CD200/CD200R, have mainly been studied in the context of their involvement in halting potentially toxic activated microglial phenotypes (Biber et al., 2007). The phenotypes embodied by the term “microglia activation” have been hotly debated. Recent nomenclatures proposed M1- and M2-like microglial phenotypes, characterized by the production of pro- or anti-inflammatory cytokines and markers, named after the alternative activation states of macrophages (Mantovani et al., 2005). The transition between these two forms, however, is not all or none and several intermediate microglia phenotypes have been described, together with the identification of overlapping features between alternatively activated phenotypes (Ponomarev et al., 2007, 2013; Olah et al., 2012; Crain et al., 2013). Furthermore, it’s now evident that no M1/M2 polarization can be supported, even for peripheral macrophages (Xue et al., 2014).

Given contemporary approaches to genome wide expression profiling, we now face the welcome prospect of devising a robust, meaningful account of microglial reactive phenotypes.

In spite of this complex picture, there is a general consensus on the relatively unique role of the CX3C system in mediating key microglial activities, mainly because of its privileged position at the interface with neurons. Microglia-neuron interaction is dynamic, as revealed by *in vivo* video microscopy using *cx3cr1^GFP/+^* mice and disclosing that microglia branches continuously survey neuronal surfaces and the cerebral microenvironment in the healthy brain, presumably to sense dysfunctional synapses, damaged neurons, or the presence of potentially dangerous agents (Davalos et al., 2005; Nimmerjahn et al., 2005). Thus, in the adult brain, microglia may exert a sentinel function for neurons and, when neuronal damage occurs, microglia rapidly react to protect or to eliminate neurons, if irreversibly damaged. CX3C signaling is deeply involved in this rescue process, directly modulating different aspects of microglia biology important for neuron protection, like the production of soluble factors directly involved in neuron survival, the modulation of phagocytic activity, but also indirectly affecting other cell types (resident or infiltrating) present in brain parenchyma that, in turn, might influence neuron survival.

CX3CR1 was localized to microglia and CX3CL1 to neurons using *in situ* hybridization, by Harrison et al. (1998). Availability of mice with a GFP fluorescent reporter for CX3CR1 transcription confirmed the former finding in adult mice (Cardona et al., 2006) and subsequent studies showed that CX3CR1 is characteristic of microglia throughout embryogenesis and during the murine lifespan (Ginhoux et al., 2010; Mizutani et al., 2012; Schulz et al., 2012). Subsequently, Kim et al. (2011) developed a CX3CL1 reporter and showed a similar neuronal distribution of CX3CL1 in all regions of the adult brain. These expression patterns are somewhat modulated but do not fundamentally alter in disease models. Therefore, CX3CL1/CX3CR1 signaling provides insight into microglial–neuronal interactions throughout the lifespan (**Table 1**) and in an immense variety of pathological conditions. This review summarizes a portion of this research.

**Table 1:** Documented effects *in (ex) vivo* of CX3CL1/CX3CR1 signaling in lifespan.

Period	Effects	Mechanisms	Reference
Embryo	Active in early microglial precursors	Unknown	Mizutani et al. (2012)
Postnatal	Circuitry refinement Cortical neuron survival Glutamatergic synapse maturation	Synaptic engulfment; timing of microglia recruitment IGF-1 production Delayed microglia-synapse interaction during development	Paolicelli et al. (2011),Hoshiko et al. (2012),Zhan et al. (2014) Ueno et al. (2013) Arnoux et al. (2013)
Adult	Dentate gyrus neurogenesis Modulation of inflammatory cytokine production	IL-1β modulation Unknown	Rogers et al. (2011), Maggi et al. (2011), Cardona et al. (2006)
	Modulation of glutamatergic neurotransmission	Post-synaptic (GluR1 dephosphorylation; NMDAR potentiation through D-Ser) modulation	Ragozzino et al. (2006), Bertollini et al. (2006), Maggi et al. (2009), Scianni et al. (2013)
	Progenitor cell proliferation in the olfactory bulb	Inflammatory cytokine production and monocyte recruitment	Blomster et al. (2011)
Aging	Dentate gyrus neurogenesis Microglia effector state	IL-1β modulation PI-3K	Bachstetter et al. (2011) Lyons et al. (2009), Wynne et al. (2010),Bachstetter et al. (2011)

## NEUROTOXICITY MODULATION: CYTOKINE AND GROWTH FACTOR PRODUCTION

The CX3CL1/CX3CR1 signaling participates in the control of production and release of several cytokines from microglia. Earlier *in vitro* studies demonstrated that LPS- and IFNγ-induced release of cytokines such as interleukin-1β (IL-1β), TNFα, 8-isoprostane, NO, and IL-6 in cultured microglia was efficiently blocked by CX3CL1 stimulation (Zujovic et al., 2000, 2001; Mizuno et al., 2003). Since then several papers confirmed and provided further *in vivo* evidence supporting the hypothesis that, together with other molecules like CD200, CD22 and CD172, CX3CL1 signaling in the brain reduces microglia reactivity to toxic stimuli, maintaining microglia in a modulated state (Biber et al., 2007). This hypothesis can be evaluated even though the status of “microglial activation” is profoundly uncertain [see (Biber et al., 2014) for a recent review].

### MODULATION OF IL-1β SIGNALING

Interleukin-1β is an inflammatory cytokine playing pivotal roles in local and systemic processes and is a key inducer of peripheral and central immune responses to infection or injury. Inhibition of IL-1β signaling has beneficial effects in a variety of experimental paradigms of acute brain damage. Despite controversial data on its neurotoxic effects *in vivo* and *in vitro*, IL-1β is considered a promising clinical target in several neuropathologies (Allan et al., 2005). In paradigms of brain injury where IL-1β increases, CX3CL1 reduces IL-1β levels, correlating with protection from damage.

Increased production of IL-1β by microglial cells isolated from the brain of *Cx3cr1^-/-^* mice upon systemic LPS challenge was described by Cardona et al. (2006). *Cx3cr1^-/-^* microglial cells from LPS-challenged mice provided a toxic insult when transplanted in the forebrain of wild type mice, resulting in neuronal TUNEL labeling. Interestingly these toxic effects were prevented eliminating IL1R signaling either with IL-1 receptor antagonist a (IL-1Ra) or in *IL1*^-/-^ mice.

Cardona et al. (2006) also showed enhanced dopaminergic cell loss in *Cx3cr1^-/-^* or *Cx3cl1^-/-^* mice after peripheral MPTP administration. Morganti et al. (2012) asked two follow-on questions: what was the role of cytokine production? and which CX3CL1 isoform (soluble or membrane-associated) was responsible for the neuroprotective effect? They reported that IL-1β signaling was blunted by soluble AAV-CX3CL1, injected in the substantia nigra pars compacts of MPTP-injected *cx3cl1*^-/-^ mice, so that less IL-1β and TNFα were produced in the ventral mesencephalon and microglial CD68 expression was reduced. These alterations in cytokine profile induced by soluble AAV-CX3CL1 correlated with reduced death of dopaminergic TH positive neurons and improved motor coordination on accelerating rotarod. A protective effect of soluble CX3CL1 on dopaminergic neurons was demonstrated by the same authors in a rat model of PD, induced by intrastriatal 6-OHDA injection (Pabon et al., 2011).

CX3CL1 decreased in the brain of aged mice possibly due to neuronal loss, and this change is accompanied by an altered effector state of microglia (Lyons et al., 2009; Wynne et al., 2010; Bachstetter et al., 2011). In aged mice, the response to systemic infection is amplified by the microglia effector response: LPS administration evokes an increased IL-1β and a reduced TGFβ expression, in comparison with young adult animals (Wynne et al., 2010).

Aging is reported to decrease neurogenesis in the hippocampal region of the brain (Kuhn et al., 1996). Similarly to aging, deficits in CX3CL1 signaling due to gene deletion or caused by CX3CR1 blocking antibodies reduced the number of progenitor cells in the hippocampal dentate gyrus (Bachstetter et al., 2011; Maggi et al., 2011). CX3CL1 administration reverted the age-related decrease in neurogenesis and chronic blockade of CX3CR1 function reduced neurogenesis and increased IL-1β in young adult mice (Bachstetter et al., 2011). IL-1β was implicated in the reduced survival and proliferation rate of neuronal progenitors in the hippocampal region of *CX3CR1^-/-^* mice, since IL-1Ra reverted the observed phenotypes (Bachstetter et al., 2011; Rogers et al., 2011).

The involvement of IL-1 in CX3CL1-induced effects in experimental models of AD is suggested by several lines of evidence. Mouse deleted of *cx3cr1* showed a strong increase of microtubule-associated protein tau (MAPT) phosphorylation upon LPS challenge, and humanized MAPT transgenic mice crossed with *cx3cr1^-/-^* mice exhibited accelerated behavioral impairment along with increased MAPT hyperphosphorylation and aggregation. These data, together with the observation that microglia-induced MAPT phosphorylation in neurons can be blocked by IL-1Ra and p38 inhibitors further supports the hypothesis that inhibiting production of IL-1β constitutes a central mechanism for CX3CL1 neuroprotective signaling (Bhaskar et al., 2010). Additional possible targets of CX3CL1 signaling in AD have been suggested: Cho et al. (2011) demonstrated that hAPP/*cx3cr1^-/-^* mice have exacerbated neuronal and cognitive function defects and increased inflammatory responses in comparison with hAPP/*cx3cr1^+/+^* mice. The lack of *cx3cr1* in this AD mouse model (hAPP-J20) did not modify plaque deposition but increased tau phosphorylation, cognitive deficits, and microglia activation, along with enhanced IL-6 and TNF-α levels. In line with these observations, AD autopsy brain sections showed reduced CX3CL1 levels in the cortex and hippocampus regions. Further supporting a potential role for CX3CR1 signaling in AD pathogenesis, Lee et al. (2010) reported that in two different mouse models of AD (APPS1 and R1.40), characterized by different velocity of β-amyloid deposition, crossing with *cx3cr1^-/-^* mice reduced the extent of amyloid deposition, the number of dystrophic neurons and of plaque-associated microglia cells. This protected phenotype was accompanied by increased CCL2 and TNF-α and elevated IL-1β expression. By contrast, a potentially toxic effect for CX3CL1 was suggested by Wu et al. (2013) demonstrating that the deficits induced by β-amyloid injection in hippocampal CA1, as well as the increased IL-1β expression and microglia activation, were all reverted by central CX3CR1 suppression by siRNA delivery. A toxic effect for CX3CR1 signaling in AD was also proposed by Fuhrmann et al. (2010) that found a reduced neuronal loss when the *3xTg AD* mice were crossed with *cx3cr1^-/-^* mice. In summary, CX3CR1/CX3CL1 signaling modifies AD-related pathology quite consistently but a unified concept of the net outcome of the effects has not been conclusively proven.

Alteration of IL-1β levels was reported in *cx3cr1-*deficient mice upon focal cerebral ischemia. Dénes et al. (2008) demonstrated a reduction in IL-1β and TNF-α production in brains of *cx3cr1^-/-^* mice upon induction of cerebral ischemia, together with reduced ischemic volume and less neuronal cell death. Similar experiments in a focal brain ischemia model were performed by Soriano et al. (2002) in *cx3cl1* deficient mouse and confirmed lower ischemic volume in the absence of CX3CL1/CX3CR1 signaling. This result was later confirmed in a pMCAO model (Cipriani et al., 2011). In apparent contrast, when CX3CL1 was exogenously administrated to *wt* mice and followed by pMCAO, a significant reduction of ischemic volume was observed while in *cx3cl1^-/-^* mice CX3CL1 administration worsened the effects of ischemia (Cipriani et al., 2011).The mechanisms underlying divergent effects of CX3CL1 administration to *wt* and *Cx3cl1^-/-^* mice remain uncertain. In favor of a protective effect of CX3CL1 in cerebral ischemia, Donohue et al. (2012) reported that stroke patients exhibited a positive correlation between high plasma CX3CL1 levels and a better clinical outcome. Interestingly, Pimentel-Coelho et al. (2013) demonstrated a protective role for CX3CR1 in neonatal hypoxic ischemia in female mice, with a possible gender specific protective effect. The authors demonstrated that hippocampal CX3CL1 was reduced by ischemia and that *Cx3cr1^-/-^* mice showed worse learning deficits and hippocampal damage after ischemia (Pimentel-Coelho et al., 2013).

In an *in vitro* genetic model of amyotrophic lateral sclerosis (ALS), where the mutated SOD1^G93A^ is specifically expressed by astrocytes, Sun et al. (2013) reported that conditioned medium from mesenchymal stem cell (MSC) cultures increased CX3CL1 and reduced TNFα, IL6, and iNOS expression on astrocytes, and mediated a protective effect towards primary motor neurons. *In vivo,* MSC administration improved the functional outcome of mutated mice. In cultured microglia, the presence of mutated SOD1^G93A^ also increased CX3CR1 levels.

Taken together, these data demonstrate that CX3CL1 attenuates inflammatory cytokine production, strongly modulating the neuroprotective activity of microglia in several brain diseases.

### PERMISSIVE EFFECTS OF ADENOSINE

Adenosine is a well-established modulator of synaptic transmission and has protective effects in the nervous system, primarily mediated through adenosine type 1 receptor (A_1_R). Adenosine is released by neurons and glial cells and its level in the brain is regulated essentially by the activity of adenosine kinase (ADK), and 5′ nucleotidase acting on extracellular ATP (Boison et al., 2010).

Upon CX3CL1 treatment of cultured microglia, increased extracellular adenosine release is observed (Lauro et al., 2008, 2010). In *in vitro* models of excitotoxicity (Lauro et al., 2010) and *in vivo* models of cerebral ischemia, the neuroprotective activity of CX3CL1 requires A_1_R activation (Cipriani et al., 2011). This neuroprotective mechanism does not derive from a direct microglia-neuron cross talk, requiring (at least *in vitro*) the involvement of astrocytes whose A_1_Rs are stimulated by adenosine released from microglia, increasing astrocyte glutamate transporter (GLT-1) expression and activity (Catalano et al., 2013).

The neuroprotective activity of adenosine has been correlated to its ability to hamper the release of excitatory glutamate at pre-synaptic terminals (Fredholm et al., 1983). In this regard, it is interesting to note that CX3CL1 has a wide spectrum of modulatory activities on glutamatergic neurotransmission, interfering with: (i) pre-synaptic glutamate release, (ii) the amplitude of AMPA currents, thereby altering GluR1 phosphorylation state (post-synaptic modulation), (iii) LTP expression and LTD induction in hippocampal CA1 region, (iv) synaptic NMDAR currents through D-serine release from glia (Meucci et al., 2000; Limatola et al., 2005; Bertollini et al., 2006; Ragozzino et al., 2006; Maggi et al., 2009; Scianni et al., 2013); (v) glutamatergic synapse maturation during development (Paolicelli et al., 2011; Arnoux et al., 2013).

All together these data demonstrate that excitatory neurotransmission, which is deeply implicated in the most common neurotoxic pathways, is affected by CX3CL1/CX3CR1 signaling, with permissive cooperativity of the adenosine system.

### GROWTH FACTORS PRODUCTION

Microglia produce a number of growth factors providing trophic support to developing or damaged brain circuits. During brain development, in the first postnatal week, CX3CR1 expression on microglia is important for the survival of cortical neurons of layer V, by regulating the production of insulin-like growth factor-1 (IGF-1; Ueno et al., 2013). In particular, increased death of layer V neurons was observed in the cerebral cortex of *cx3cr1^GFP/GFP^* mice, in which microglia produced a significantly reduced amount of the growth factor IGF-1. So during brain development, CX3CR1-mediated IGF-1 secretion from microglia modulates neuronal survival in postnatal cortical layer V.

A specific BDNF polymorphism (Val66Met) is associated with memory deficits and increased vulnerability to anxiety and depressive disorders both in humans and mice. Interestingly, this polymorphism also associates with impaired CX3CL1/CX3CR1 hippocampal signaling, reducing expression of CX3CL1 protein and mRNA in dorsal hippocampus (Wang et al., 2014). Chronic CX3CL1 infusion in the hippocampal region of *Val66Met* mice recovered memory deficits, restored neurogenesis in the DG and increased Akt phosphorylation levels.

## NEUROTOXICITY MODULATION: MICROGLIA PHAGOCYTIC ACTIVITY

One of the most intriguing functions of microglia in the central nervous system is certainly its activity as the resident phagocyte. In development and in pathological neuroinflammatory conditions, microglial phagocytosis is important for the elimination of dead and damaged neurons, myelin residues and β-amyloid peptides [reviewed in (Sierra et al., 2013)]. During normal brain development, microglial phagocytosis is involved in eliminating supernumerary neurons, but also in synaptic pruning and circuit refinement (Sierra et al., 2010; Tremblay et al., 2010). Among the key factors that emerged as potential modulators of neuron phagocytosis by microglia during development is the complement system, with the proteins C1q and C3 expressed on synapses and microglial CR3/CD11b mediating the elimination of immature and weakly active synapses (Schafer et al., 2012). TGF-β produced by astrocytes signals to retinal ganglion cells for C1q expression (Bialas and Stevens, 2013), thus favoring retinogeniculate refining, and inserting astrocytes in microglia-neuron communication.

Cell damage causes high-grade ATP release (both from damaged cells and through astrocyte exocytosis) regulating microglial process extension (Davalos et al., 2005) and also contributing to inflammasome activation within microglia (Ransohoff and Brown, 2012). Under stress, neurons manifest the phosphatidylserine (PS) “eat me” signal which can be recognized by several microglia receptor/adaptor complexes including Gas6/MerTK and MFGE8/vitronectin receptor (De Simone et al., 2004; Elliott et al., 2009). In ischemic conditions potentially viable neurons reversibly express PS on their surface and are vulnerable to be taken up and destroyed by microglia (Neher et al., 2011). Selective elimination of MFGE8/vitronectin receptor “eat me” signaling strongly improved experimental stroke outcome (Neher et al., 2013).

CX3CR1 signaling also modulates microglia phagocytosis of neurons and their processes in both physiological and pathological conditions. During development, CX3CL1/CX3CR1 signaling contributes to refinement of synaptic elements in varied CNS regions during the postnatal “critical period” (Tremblay et al., 2010; Paolicelli et al., 2011; Hoshiko et al., 2012). This fine-tuning of anatomical connections is important to establish and build an optimal functional circuitry (Zhan et al., 2014).

In Alzheimer’s disease (AD), CX3CL1/CX3CR1 blockade modulates microglia phagocytic activity in a way that markedly attenuates amyloid deposition (Lee et al., 2010; Liu et al., 2010). In particular, CX3CR1 deficient microglia show increased phagocytic activity when crossed with different AD mouse models CRND8 (Liu et al., 2010) or APPPS1 and R1.40 (Lee et al., 2010), resulting in attenuated Aβ deposition. Based on results that look beyond amyloid deposition in various models, the overall outcome of eliminating CX3CR1 signaling remains in doubt (Bhaskar et al., 2010; Fuhrmann et al., 2010; Cho et al., 2011).

Neurons damaged by toxic insults, like glutamate, release more CX3CL1 that increases MFG-E8 expression on microglia (Leonardi-Essmann et al., 2005; Fuller and Van Eldik, 2008; Noda et al., 2011). As noted above, MFG-E8 is a PS receptor that recognize PS on injured neurons and acts as an adaptor recognized by microglial vitronectin receptor, inducing damaged-cell uptake. CX3CL1 up-regulation of MFG-E8 expression was associated to increased heme oxygenase 1 (HO-1) expression through the MAPKs ERK and JNK and the nuclear factor erythroid 2 related factor (NFE2LE). Lastres-Becker et al. (2014) reported that CX3CL1 reduced Tau-induced microgliosis via up-regulating transcription factor NRF2/NFE2LE, along with increased HO-1 expression. Since the increased expression of HO-1 is reported to mediate anti inflammatory activities (Chora et al., 2007), this pathway could be involved in the reported neuroprotective effects of CX3CL1 in different Tau pathology models (Nash et al., 2013).

These data indicate that the clearance and phagocytic activity of microglia can be modulated by CX3CL1 signaling through varied mechanisms, and that the final outcome in term of neuroprotection or neurotoxicity might result from the presence of co-stimulatory signals and, consequently, from the effector programs of microglia.

## NEUROTOXICITY MODULATION: EFFECTS ON NEURAL PRECURSORS

*In vitro*, CX3CL1 promoted survival of neural precursor cells upon growth factor withdrawal from the culture medium (Krathwohl and Kaiser, 2004). Further, *cx3cr1^-/-^* mice have reduced neurogenesis in the hippocampal DG region (Bachstetter et al., 2011; Maggi et al., 2011) but this reduction was accompanied by contrasting effects on memory deficits and synaptic plasticity: Maggi et al., (Maggi et al., 2011) reported that *cx3cr1^-/-^* mice had increased hippocampal LTP and performed better in the Morris water maze test, while, in the same mouse strain, Rogers et al. (2011) described reduced hippocampal LTP and deficits in the Morris water maze. At least in part, these conflicting data reflect the lack of simple relations between DG neurogenesis, plasticity processes in the hippocampal CA1 region and learning behavior.

More recently, Vukovic et al. (2012) demonstrated that CX3CR1 deficiency impairs neurogenesis both in young and aged mice and that voluntary physical exercise increased brain CX3CL1 levels and DG neurogenesis, both effects being abolished by intrahippocampal CX3CR1 antibody injection.

In the olfactory bulb, CX3CR1 deletion increased olfactory sensory neuron death upon bulbectomy and *cx3cr1^-/-^* mice showed reduced proliferation of intraepithelial stem progenitor cells, increased macrophage recruitment in the lesioned epithelium and increased TNF-α and IL-6 levels (Blomster et al., 2011).

All together these data suggest that neuroprotective effects of CX3CL1 might arise partly from an increased proliferation or survival of progenitor elements in selected brain regions where neurogenesis continues throughout adult life.

## NEUROTOXICITY MODULATION: INDIRECT EFFECTS

### MODULATION OF ASTROCYTE ACTIVITY

In the CNS, the unique expression of CX3CR1 on microglia clearly suggests that the neuroprotective effects of CX3CL1 depend primarily on the direct communication between microglia and neurons. Recent evidence suggested that CX3CL1/CX3CR1 signaling in neuroprotection again excitotoxic insult might not be restricted to a direct microglia-neuron communication but also involves indirectly modulation of astrocyte activity. Catalano et al. (2013), in fact, demonstrated that CX3CL1, acting on microglia, induced the production and release of soluble factors that exerted their effects on astrocytes, inducing the functional up regulation and the increased expression of the excitatory amino acid transporter GLT-1. As already reported in *in vitro* and *in vivo* systems (Lauro et al., 2010; Cipriani et al., 2011), this cross-talk requires the “permissive” presence of adenosine, specifically acting on astrocyte A_1_R (Catalano et al., 2013). These data demonstrated for the first time a role for astrocytes in mediating the neuroprotection induced by CX3CL1/CX3CR1 signaling between microglia and neurons.

### EFFECTS THROUGH LEUKOCYTE RECRUITMENT

Neuroprotective effects of CX3CL1 could also arise from hematogenous leukocytes that enter the brain upon specific, injury-induced challenge.

In mouse experimental allergic encephalomyelitis (EAE) Huang et al. (2006) demonstrated that CX3CR1 deficient mice had an exacerbated phenotype, with severe spastic paralysis and hemorrhagic inflammation in the CNS, resulting in higher mortality. The increased toxicity resulted from an altered control of NK cells entry into the brain of EAE-affected animals. These findings were extended by Garcia et al. (2013), who showed that CX3CR1 deficiency limited to bone marrow cells of mice with EAE resulted in more severe pathology, with increased demyelination, axonal damage and reduced calbindin positive neurons in the cerebellum. These effects correlated with increased CD115^+^, Ly6C^low^CD11c^+^ dendritic cell infiltration in the CNS and increased IL-17 and IFNγ expression in the cerebellum, forebrain and spinal cord.

Mills et al. (2008) reported that in *CD73^-/-^* mice, where the ectonucleotidase responsible for extracellular adenosine accumulation was absent, EAE induction produced a milder phenotype. More recently, the same group (Mills et al., 2012) reported that extracellular adenosine increased CX3CL1 expression and that CX3CL1 promoted lymphocyte entry into the brain of EAE mice. Therefore, in CD73 deficient mice, extracellular adenosine cannot be produced, CX3CL1 is not upregulated and a reduced amount of lymphocytes enter cerebral parenchyma, resulting in a protected phenotype (Mills et al., 2012).

In focal cerebral ischemia, Dénes et al. (2008) reported a reduced leukocyte (CD45^+^ cells) infiltration in the brain of *cx3cr1^-/-^* mice and a consistently reduced damaging of the blood brain barrier. This correlated with a protected phenotype, with reduced ischemic volume and lesser numbers of apoptotic cells and a better performance in the behavioral test of adhesive tape removal.

All together these data highlight that CX3CL1/CX3CR1 signaling modulates the entry of hematogenous leukocytes, with beneficial or deleterious consequences contingent on context.

## CONCLUSION

This review highlights the manifold effects of altering CX3CR1/CX3CL1 signaling, which can be observed throughout the lifespan from early embryogenesis through diseases of the aging brain. For the most part, CX3CR1/CX3CL1 can be viewed as a fulcrum with which to modify microglial physiology to probe into the panoply of functions of these versatile and essential cells. Adding to complexity but also lending heuristic value, the effects of modifying CX3CR1/CX3CL1 pathways are extremely context dependent. Nevertheless in each case insights about upstream and downstream signaling from CX3CR1/CX3CL1 have been informative and may point to new therapeutic directions for brain disease. Finally, it is now clear that macroglia (such as astrocytes) and hematogenous leukocytes also contribute to the varied physiology of the CX3CR1/CX3CL1 signaling system.

## Conflict of Interest Statement

The authors declare that the research was conducted in the absence of any commercial or financial relationships that could be construed as a potential conflict of interest.
